# Supplementary material for: Glycemic variability and all-cause mortality in a large prospective southern European cohort of patients with differences in glycemic status
Source: PLoS One. 2022 Jul 25;17(7):e0271632. doi: 10.1371/journal.pone.0271632 (PMC9312379; doi:10.1371/journal.pone.0271632)
Supplement: Attachment — Submitted filename: 2022_06_01_Rebutal letter.docx [file pone.0271632.s001.docx]

**Rebuttal letter**

We thank the editor and the two reviewers for their comments on our manuscript entitled “*Glycemic variability and all-cause mortality in a large prospective southern European cohort of patients with differences in glycemic status* “. Below is our response to each point raised by the academic editor and reviewers.

We hope that we satisfyingly addressed them, and that the manuscript will be now suited for publication.

Sincerely,

Salinero-Fort MA

On behalf of all authors,

**Academic editor:**

**1. Please ensure that your manuscript meets PLOS ONE's style requirements, including those for file naming. The PLOS ONE style templates can be found at**

https://journals.plos.org/plosone/s/file?id=wjVg/PLOSOne_formatting_sample_main_body.pdf and

<https://journals.plos.org/plosone/s/file?id=ba62/PLOSOne_formatting_sample_title_authors_affiliations.pdf>.

File naming was edited to comply with the style requirements. We hopefully have no divergences from the style requirements now.

**2. Please provide additional details regarding participant consent. In the Methods section, please ensure that you have specified (1) whether consent was informed and (2) what type you obtained (for instance, written or verbal). If your study included minors, state whether you obtained consent from parents or guardians. If the need for consent was waived by the ethics committee, please include this information.**

In methods section include the following paragraph:

*“The study was conducted according to the guidelines of the Declaration of Helsinki and approved by the Institutional Review Board of Ramón y Cajal Hospital (Madrid) for the MADIABETES cohort (approval identification code:2017/335) and the Institutional Review Board of Carlos III Hospital (Madrid) for the SPREDIA cohort (approval identification code: P07/2012). A written informed consent was obtained from all subjects involved in the study”.*

The e-mail of Institutional Review Board of Ramón y Cajal Hospital (Madrid) is: ceic.hrc@salud.madrid.org

**3. Thank you for stating the following financial disclosure:**

**“This study was funded by Instituto de Salud Carlos III through projects “PI15/00259” and “PI18/01025” and co-funded by the European Regional Development Fund, “A way of shaping Europe””**

**Please state what role the funders took in the study.  If the funders had no role, please state: ""The funders had no role in study design, data collection and analysis, decision to publish, or preparation of the manuscript.""**

**If this statement is not correct you must amend it as needed.**

**Please include this amended Role of Funder statement in your cover letter; we will change the online submission form on your behalf.**

We confirm that:

The funders had no role in study design, data collection and analysis, decision to publish, or preparation of the manuscript.

**4. Thank you for stating the following in your Competing Interests section:**

**“The authors declare that they have no known competing financial interests or personal relationships that could influence the work reported in this paper.”**

**Please complete your Competing Interests on the online submission form to state any Competing Interests. If you have no competing interests, please state ""The authors have declared that no competing interests exist."", as detailed online in our guide for authors at http://journals.plos.org/plosone/s/submit-now**

**This information should be included in your cover letter; we will change the online submission form on your behalf.**

We confirm that:

The authors have declared that no competing interests exist

**5. In your Data Availability statement, you have not specified where the minimal data set underlying the results described in your manuscript can be found. PLOS defines a study's minimal data set as the underlying data used to reach the conclusions drawn in the manuscript and any additional data required to replicate the reported study findings in their entirety. All PLOS journals require that the minimal data set be made fully available. For more information about our data policy, please see http://journals.plos.org/plosone/s/data-availability.**

**Upon re-submitting your revised manuscript, please upload your study’s minimal underlying data set as either Supporting Information files or to a stable, public repository and include the relevant URLs, DOIs, or accession numbers within your revised cover letter. For a list of acceptable repositories, please see http://journals.plos.org/plosone/s/data-availability#loc-recommended-repositories. Any potentially identifying patient information must be fully anonymized.**

**Important: If there are ethical or legal restrictions to sharing your data publicly, please explain these restrictions in detail. Please see our guidelines for more information on what we consider unacceptable restrictions to publicly sharing data: http://journals.plos.org/plosone/s/data-availability#loc-unacceptable-data-access-restrictions. Note that it is not acceptable for the authors to be the sole named individuals responsible for ensuring data access.**

**We will update your Data Availability statement to reflect the information you provide in your cover letter.**

There are restrictions on the availability of data for the MADIABETES study, due to the signed consent agreements around data sharing, which only allow access to external researchers for research following the project purposes.

Requestors wishing to access the MADIABETES data used in this study can request it to the MADIABETES Steering Committee: [estudios.fiibap@salud.madrid.org](mailto:estudios.fiibap@salud.madrid.org)

The request will then be passed to members of the MADIABETES Steering Committee for deliberation.

However, the minimal data set is available from the Institutional Repository of the Regional Health System of the Community of Madrid (<https://repositoriosaludmadrid.es/>)

<https://repositoriosaludmadrid.es/handle/20.500.12530/326/browse?type=author&order=ASC&rpp=20&authority=e55f8892-3241-4a96-a0d6-640436d5c75e&value=SALINERO-FORT%2C+MIGUEL+A>.

**6. We note that you have indicated that data from this study are available upon request. PLOS only allows data to be available upon request if there are legal or ethical restrictions on sharing data publicly. For more information on unacceptable data access restrictions, please see http://journals.plos.org/plosone/s/data-availability#loc-unacceptable-data-access-restrictions.**

**In your revised cover letter, please address the following prompts:**

**a) If there are ethical or legal restrictions on sharing a de-identified data set, please explain them in detail (e.g., data contain potentially sensitive information, data are owned by a third-party organization, etc.) and who has imposed them (e.g., an ethics committee). Please also provide contact information for a data access committee, ethics committee, or other institutional body to which data requests may be sent.**

**b) If there are no restrictions, please upload the minimal anonymized data set necessary to replicate your study findings as either Supporting Information files or to a stable, public repository and provide us with the relevant URLs, DOIs, or accession numbers. For a list of acceptable repositories, please see http://journals.plos.org/plosone/s/data-availability#loc-recommended-repositories.**

**We will update your Data Availability statement on your behalf to reflect the information you provide.**

There are restrictions on the availability of data for the MADIABETES study, due to the signed consent agreements around data sharing, which only allow access to external researchers for research following the project purposes.

Requestors wishing to access the MADIABETES data used in this study can request it to the MADIABETES Steering Committee: [estudios.fiibap@salud.madrid.org](mailto:estudios.fiibap@salud.madrid.org)

The request will then be passed to members of the MADIABETES Steering Committee for deliberation.

**7. Please note that in order to use the direct billing option the corresponding author must be affiliated with the chosen institute. Please either amend your manuscript to change the affiliation or corresponding author, or email us at plosone@plos.org with a request to remove this option.**

Corresponding author:

Salinero-Fort MA^1-5^

1. Foundation for Research and Biomedical Innovation of Primary Care of the Community of Madrid (FIIBAP) [payment institution]
2. The Hospital La Paz Institute for Health Research (IdiPAZ).
3. Health Services and Chronic Conditions Research Network (REDISSEC), Madrid, Spain.
4. General Subdirectorate of Research and Documentation. Department of Health, Madrid, Spain.
5. Alfonso X El Sabio University, Madrid, Spain.

**8. One of the noted authors is a group or consortium [Abanades-Herranz JC]. In addition to naming the author group, please list the individual authors and affiliations within this group in the acknowledgments section of your manuscript. Please also indicate clearly a lead author for this group along with a contact email address.**

The MADIABETES consortium is currently led by Dr. Iriarte-Campo V, a researcher at the Foundation for Research and Biomedical Innovation of Primary Care of the Community of Madrid (FIIBAP). His e-mail is: [victor.iriarte@salud.madrid.org](mailto:victor.iriarte@salud.madrid.org)

Dr. Abanades-Herranz since 2020 is not the leader or head of the group.

**Reviewer #1: The authors have found in their study that CV-FPG is useful for measurement of GV. Although the finding are potential, reviewer has few concerns:**

**-data representation should be re-arranged. For instance, few table are too long to read. Split them into two.**

As suggested by the reviewer, the previous table 1 is now broken down into two new tables and the previous table 3 is now broken down into three new tables. This will surely make the tables easier to read.

**- Consult the statistician to confirm the statistical tests used in the study.**

The normality of the distribution of all the continuous variables was checked using the Kolmogorov-Smirnov Test and all of them were normally distributed, so parametric tests could be used.

Also, Valentín Hernández-Barrera, an expert statistician of Preventive Medicine and Public Health Teaching and Research Unit, Health Sciences Faculty, Universidad Rey Juan Carlos, Madrid, Spain (ORCID: 0000-0001-5790-1959; E-mail: [valentin.hernandez@urjc.es](mailto:valentin.hernandez@urjc.es)) confirms that the statistical tests used are correct and appropriate. Several previous studies, with similar designs to ours, have used the same statistical methods applied in our investigation:

- Kim C, Sohn J-H, Jang MU, Kim S-H, Choi M-G, Ryu O-H, et al. (2015) Association between Visit-to-Visit Glucose Variability and Cognitive Function in Aged Type 2 Diabetic Patients: A Cross-Sectional Study. PLoS ONE 10(7): e0132118.doi:10.1371/journal.pone.0132118
- Wang A, Liu X, Xu J, Han X, Su Z, Chen S, Zhang N, Wu S, Wang Y, Wang Y. Visit-to-Visit Variability of Fasting Plasma Glucose and the Risk of Cardiovascular Disease and All-Cause Mortality in the General Population. J Am Heart Assoc. 2017 Nov 29;6(12):e006757. doi: 10.1161/JAHA.117.006757. PMID: 29187392; PMCID: PMC5779006.
- Xu D, Fang H, Xu W, Yan Y, Liu Y, Yao B. Fasting plasma glucose variability and all-cause mortality among type 2 diabetes patients: a dynamic cohort study in Shanghai, China. Sci Rep. 2016 Dec 22;6:39633. doi: 10.1038/srep39633. PMID: 28004765; PMCID: PMC5177938.

Lastly, Hans DeVries J (Academic Medical Center at the University of Amsterdam, Amsterdam, the Netherlands) in his article entitled “Glucose Variability: Where It Is Important and How to Measure It” (Diabetes. 2013 May;62(5):1405-8. doi: 10.2337/db12-1610. PMID: 23613566), says: “*When diabetes investigators want to assess glucose variability, I would recommend coefficient of variation and mean absolute glucose (MAG)*”.

**- Check for the grammar and typo error.**

An expert native translator has reviewed the manuscript and confirms that there are no grammatical errors. Please find enclosed a certificate from the translator.

-**Please report the other medical underlyig conditions and how they affect the finding?**

Our study has incorporated the main variables that can affect glycemic variability in the multivariate analysis. Study's covariates are as follows: Age, sex, cardiovascular disease, hypertension, use of aspirin, statins, GLP-1 receptor agonists, SGLT-2 inhibitors and DPP-4 inhibitors, baseline FPG and average HbA1c (when at least two measurements)

The studies listed below have used the same or similar control variables as our study.

- Cardoso CRL, Leite NC, Moram CBM, Salles GF. Long-term visit-to-visit glycemic variability as predictor of micro- and macrovascular complications in patients with type 2 diabetes: The Rio de Janeiro Type 2 Diabetes Cohort Study. Cardiovasc Diabetol. 2018 Feb 24;17(1):33. doi: 10.1186/s12933-018-0677-0. PMID: 29477146; PMCID: PMC6389075.
- Orsi E, Solini A, Bonora E, Fondelli C, Trevisan R, Vedovato M, Cavalot F, Gruden G, Morano S, Nicolucci A, Penno G, Pugliese G; Renal Insufficiency and Cardiovascular Events (RIACE) Study Group. Haemoglobin A1c variability is a strong, independent predictor of all-cause mortality in patients with type 2 diabetes. Diabetes Obes Metab. 2018 Aug;20(8):1885-1893. doi: 10.1111/dom.13306. Epub 2018 Apr 19. PMID: 29582548.

An alternative option would have been to use propensity score matching, but this was discarded for two reasons:

1) No studies have ever used the propensity score to measure the effect of glycemic variability on mortality as can be demonstrated by the following search strategy in PubMed: "glycemic variability" AND "propensity score matching": 0 Results..
2) In the limitations section, we have added the following explanation about the not use of propensity score matching.

*“Given the observational nature of the present study, individuals with higher GV and lower GV were dissimilar. Therefore, to obtain an accurate picture of the association between GV and all-cause mortality, it was necessary to adjust for differences in both groups in the multivariate analysis. Propensity score matching (PSM) would be a more appropriate alternative that would yield less biased results than standard methods such as Cox regression. However, one of the drawbacks of PSM is the loss of sample in terms of size. In addition, PSM should not be used in practice because our sample size was insufficiently large. Given that propensity scores can only control for observed confounders, they cannot be counted upon to balance unobserved covariates”*

**Reviewer #2: The current study “Glycemic variability and all-cause mortality in a large prospective southern European cohort of patients with differences in glycemic status” contributes in highlighting the significance to include glycemic variability (GV) as one of the factors to monitor the prognosis of different glycemic status patients.

The finding do suggest how GV could behave as a long-term predictor of all-cause mortality in individuals with certain underlying conditions with variation included .It is rightly understood, with the limitation of the method to satisfy all the variables not limited to duration of the disease, diabetes treatments, or microalbuminuria etc, which would prevent this to be used with confidence.**

**But the authors do make a valid case for inclusion of GV analysis through their proposed methods so as to derive information for managing the outcome of the diabetic condition of the patients.**

The methodological approach to studying the effect of glycemic variability on mortality is similar to that proposed by all the studies published to date. The ideal would have been to ensure that both groups: those exposed to greater glycemic variability and those exposed to lesser glycemic variability, were similar in all the variables predictive of mortality. This approach would have needed to perform a propensity score matching, which was discarded because it would have meant a considerable reduction in the study sample. To date, no article like ours has done so.

**There are however, some concerns with the current draft of the manuscript. The authors should follow a consistent mode of representation. E.g. at some places Fig. is used while at some places Figure is written. Kindly adhere to one form of writing.**

Thank you for this comment. Sorry for the lack of consistency. Following your suggestion, the text has been completely reviewed and all these errors edited.
